# Redox Imbalance in Neurological Disorders in Adults and Children

**DOI:** 10.3390/antiox12040965

**Published:** 2023-04-20

**Authors:** Federica Rey, Clarissa Berardo, Erika Maghraby, Alessia Mauri, Letizia Messa, Letizia Esposito, Giovanna Casili, Sara Ottolenghi, Eleonora Bonaventura, Salvatore Cuzzocrea, Gianvincenzo Zuccotti, Davide Tonduti, Emanuela Esposito, Irene Paterniti, Cristina Cereda, Stephana Carelli

**Affiliations:** 1 Pediatric Clinical Research Center “Romeo ed Enrica Invernizzi”, Department of Biomedical and Clinical Sciences, University of Milano, 20157 Milano, Italy; 2 Center of Functional Genomics and Rare diseases, Department of Pediatrics, Buzzi Children’s Hospital, 20154 Milano, Italy; 3 Department of Biology and Biotechnology “L. Spallanzani”, University of Pavia, 27100 Pavia, Italy; 4 Department of Electronics, Information and Bioengineering (DEIB), Politecnico di Milano, 20133 Milano, Italy; 5 Department of Chemical, Biological, Pharmaceutical and Environmental Sciences, University of Messina, 98166 Messina, Italy; 6 Department of Medicine and Surgery, University of Milano Bicocca, 20126 Milano, Italy; 7 Child Neurology Unit, Buzzi Children’s Hospital, 20154 Milano, Italy; 8 Center for Diagnosis and Treatment of Leukodystrophies and Genetic Leukoencephalopathies (COALA), Buzzi Children’s Hospital, 20154 Milano, Italy; 9 Department of Pediatrics, Buzzi Children’s Hospital, 20154 Milano, Italy

**Keywords:** oxygen, redox, neurodegenerative diseases, neurodevelopmental disorders, Alzheimer’s disease, Parkinson’s disease, amyotrophic lateral sclerosis, X-adrenoleukodystrophies, spinal muscular atrophy, mucopolysaccharidoses, Pelizaeus–Merzbacher disease

## Abstract

Oxygen is a central molecule for numerous metabolic and cytophysiological processes, and, indeed, its imbalance can lead to numerous pathological consequences. In the human body, the brain is an aerobic organ and for this reason, it is very sensitive to oxygen equilibrium. The consequences of oxygen imbalance are especially devastating when occurring in this organ. Indeed, oxygen imbalance can lead to hypoxia, hyperoxia, protein misfolding, mitochondria dysfunction, alterations in heme metabolism and neuroinflammation. Consequently, these dysfunctions can cause numerous neurological alterations, both in the pediatric life and in the adult ages. These disorders share numerous common pathways, most of which are consequent to redox imbalance. In this review, we will focus on the dysfunctions present in neurodegenerative disorders (specifically Alzheimer’s disease, Parkinson’s disease and amyotrophic lateral sclerosis) and pediatric neurological disorders (X-adrenoleukodystrophies, spinal muscular atrophy, mucopolysaccharidoses and Pelizaeus–Merzbacher Disease), highlighting their underlining dysfunction in redox and identifying potential therapeutic strategies.

## 1. Introduction

Oxygen is a molecule that is fundamental for life, as its action in numerous cellular processes can alter gene expression and lead to pathways’ function or dysfunction [1]. The cellular mechanisms that involve an oxidation-reduction reaction are termed redox signaling, and these are the key processes that govern most aspects of a cell’s life [2].

**Figure 1:**
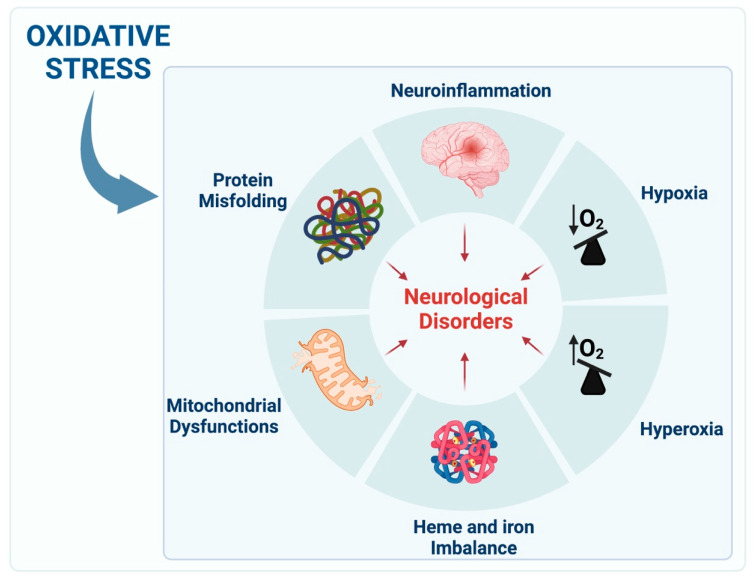
Schematic representation of the alterations in redox mechanisms that can lead to numerous aberrant processes, and ultimately lead to neurological disorders.

The brain is an entirely aerobic organ, and for this reason, it is extremely sensitive to oxygen balance [3]. The resting metabolic rate is defined as the energy needed by the organism when at rest, and 20–25% of this energy is required for brain functioning [3]. Indeed, to maintain the ionic equilibria and neurotransmitters uptake necessary for neuronal communication, there is the need for a high rate of ATP formation and consumption [3]. Oxygen storage in the brain is limited, and without a blood supply, cerebral metabolism would be sustained for only 1 s [3,4]. The condition of low oxygen levels is known as hypoxia [5]. On the contrary, a state of excess supply of O_2_ in tissues and organs is known as hyperoxia [6]. Aberrations in oxygen levels can often lead to pathological states, and hypoxia is a primary cause of clinical impairment in stroke and traumatic brain injury [1,3]. Processes that are characterized by alterations in oxygen metabolism and redox signaling can be the primary causes of neurological diseases [7]. These are strictly related to the production of radical oxygen species (ROS), mitochondria abnormalities, protein misfolding and neuroinflammation [8]. Remarkably, aberrations in these processes can lead to the insurgence of neurological disorders (Figure 1).

## 2. Cellular Pathways of Redox Imbalance

Redox signaling processes concern all these pathways, which present an increase or decrease of reactive species [9]. Herein, we present a brief overview of the main cellular pathways affected by these processes, which will be subsequently addressed more in-depth for their correlation with the pathogenesis of neurological disorders.

### 2.1. Hypoxia

Hypoxia is defined as a condition of decreased oxygen levels, and it is a common pathophysiological condition that can affect brain metabolism [6]. With long term exposure to hypoxia, the mitochondrial volume density decreases and the expression of the electron transport chain complexes is decreased, with a subsequent effect on neuronal metabolism [10]. A significative increase of the exploration of oxygen sensing started from the discovery of erythropoietin (EPO), a kidney-derived glycoprotein mainly induced in hypoxia conditions by both hypoxia-inducible factor-1 and -2 (HIF-1 and HIF-2, respectively) [11]. EPO has been found to mediate its neuroprotective effect through its receptor EPOR, especially in hypoxic conditions [6,12,13]. Indeed, even if the roles of EPO are not limited to the brain, its action in this organ is surely fundamental, as it has been found to promote and stimulate neurogenesis [14,15,16]. Its neuroprotective role can be also correlated with its action as an inhibitor of ROS production, as well as its function in counteracting oxidative stress [11].

Mild or important cognitive impairment is also known to be a consequence of the hypoxic damage that takes place during neurological impairments, such as ischemic strokes and traumatic brain injuries. The role of the relative hyperoxia induced by reperfusion in those cases is an interesting subject of ongoing studies [17,18]. Interestingly, oxidative damage due to cervical spinal cord injury (SCI) can also exacerbate muscle atrophy and weakness in the disease. In this case, hyperbaric oxygen and subsequent ROS generation has been found to stimulate adaptations of the diaphragm oxidative capacity, and this results in a reduction of oxidative stress and inflammation [19].

An important aspect to consider when describing hypoxia is the biochemical mechanism that leads to redox imbalance. Specifically, if the oxygen supply becomes inadequate, the tricarboxylic acid cycle’s (TCA) functions are inhibited and pyruvate derived from glycolysis accumulates [20]. In this condition, pyruvate is converted to lactate through a reaction that provides the regeneration of nicotinamide adenine dinucleotide (NAD) for the continuance of glycolysis [21]. Since the ratio of lactate to pyruvate increases, this ratio has been proposed as a mechanism for assessing hypoxia [20]. Glycolysis produces ATP from ADP but in an inefficient way, as 1 mole of glucose-6-phosphate metabolized to pyruvate produces just 2 moles of ATP instead of the 36 moles that could be produced by the subsequent metabolism of pyruvate in the TCA [22]. All of these events culminate with the failure of electrons transport and the subsequent accumulation of hydrogen ions, which cause redox imbalance and tissue acidosis [23].

### 2.2. Hyperoxia and ROS Production

Opposite to hypoxia, hyperoxia is defined as a condition of increased oxygen levels. It stimulates ROS formation, such as hydrogen peroxide, hydroxyl radicals and superoxide anions, which can affect signaling pathways by directly modifying regulatory proteins through carbonylation, formation of disulphide bonds, glutathionylation and nitrosylation [24]. Molecular oxygen has two unpaired electrons in separate orbitals in its outer electron shell. An increase in oxygen availability leads to incomplete reduction of O_2_, which produces O_2_^•−^, HO^•^ and H_2_O_2_. Although ROS are essential regulators of multiple physiological functions of living organisms, such as signal transduction and gene transcription, their imbalance is a major cause of oxidative damage to cellular macromolecules, resulting in numerous neuronal pathologies [25].

ROS are produced by multiple cellular processes and can be overproduced in response to different stimuli, including xenobiotic compounds, cytokines and bacterial invasion [26]. Mitochondria represent major sources in ROS production due to their role in oxidative ATP production. Indeed, the superoxide radical (O_2_^•−^) is produced in various sites of the mitochondria, such as complex I and complex III, which release this molecule into the mitochondrial matrix (MM) [27]. Manganese superoxide dismutase (Mn-SOD) then converts the O_2_^•−^ superoxide radical in MM to hydrogen peroxide (H_2_O_2_), which can be further converted by mitochondrial aconitase to a hydroxyl radical (^•^OH) via a Fenton reaction [28]. Other intracellular structures involved in the production of ROS are the peroxisomes and the endoplasmatic reticulum (ER), which generate a wide range of ROS and RNS. Specifically, peroxisomes produce O_2_^•−^ in both the matrix and membrane through the intervention of two enzymes: xanthine oxidoreductase (XOR) and urate oxidase (UO) [29]. XOR catalyzes the formation of uric acid during purine metabolism, which is further converted to allantoin by UO [30]. Regarding the role of ER in ROS production, it has been demonstrated that the accumulation of misfolded proteins in ER during hyperoxia initiates the unfolded protein response (UPR) [31]. UPR triggers ROS production, which can promote ER stress through the intervention of endoplasmic reticulum oxidoreductin-1 (ERO1). ERO1 catalyzes a double reaction because it leads to the production of H_2_O_2_ and the conversion of glutathione (GSH) to glutathione disulfide (GSSG) [32]. Accumulation of both H_2_O_2_ and oxidized glutathione further increase ER stress. The ratio between GSH and GSSG is an essential indicator of the redox status in the ER lumen [33].

### 2.3. Role of the Antioxidant System of Cells in Redox Balance

The antioxidant system is fundamental to maintain cellular homeostasis and counteract oxidative stress. The antioxidant system works by exploiting two mechanisms: non-enzymatic and enzymatic [34]. Among the non-enzymatic defenses, GSH and α-lipoic acid (ALA) must be mentioned. GSH is a thiol tripeptide formed by L-γ-glutamyl-L-cysteinyl-glycine, and is present in every cell, especially in the brain [35]. Besides its scavenging role, GSH assists several processes, such as cellular differentiation and proliferation, apoptosis and neurotransmission [36]. Intracellular glutathione is mostly present in its reduced form, even if it can be oxidized into GSSG by glutathione peroxidase (GPx), while the inverse conversion is performed by the glutathione reductase (GR) [37]. With respect to other organelles, mitochondria contain higher levels of GSH: even if they lack the enzymes required by its biosynthesis, they are able import the tripeptide thanks to dicarboxylate and 2-oxogluratate carriers [38]. Under physiological conditions, the ratio of GSH to GSSH hangs in favor of GSH; on the contrary, in the presence of oxidative stress, such as in neurological disorders, the GSH/GSSH ratio sharply decreases. In this context, significant evidence has been reported that the GSH levels in the blood and brain decreased in AD patients, as well as in PD, ALS and autism patients [36]. Interestingly, CNS cells show a different sensitivity to oxidative stress based on their GSH mitochondrial concentration; in fact, astrocytes are more vulnerable to oxidative stress [39].

ALA is an endogenous antioxidant that contains two thiol groups. It is naturally synthesized in mitochondria, where it functions as a cofactor in mitochondrial metabolism and biogenesis, but it can be supplemented by food [40]. Differently from glutathione, in which only the reduced form acts as an antioxidant, in this case, both the oxidized form (LA) and the reduced form of dihydrolipoic acid (DHLA) can neutralize free radicals, chelating heavy metals. Moreover, DHLA can regenerate other antioxidant molecules with low molecular weight, such as GSH. In addition, ALA exhibits anti-inflammatory properties, reducing proinflammatory mediators, such as TNF-α, and increasing anti-inflammatory cytokines, such as IL-10 [41]. Thanks to ALA’s amphiphilic properties, it can easily cross the blood–brain barrier, leading to beneficial effects in the CNS. In rat models of PD, it has been demonstrated that ALA was able to reduce 6-OHDA and H_2_O_2_, along with nitric oxide, preventing neuronal damage [42].

Besides the GPx mentioned before, superoxide dismutase (SOD) and catalase (CAT) also belong to the first line of enzymatic antioxidant defenses. Three types of SODs can be distinguished in humans, depending on the metal cofactors to which they bind: SOD1, present in the cytosol and in the mitochondrial intermembrane, binds to copper/zinc (Cu/Zn); SOD2 is present at the mitochondrial level and interacts with manganese (Mn); and SOD3, the extracellular form, complexed with copper and zinc [43]. SODs catalyze a dismutase reaction, converting highly reactive superoxide radicals into hydrogen peroxide and molecular oxygen, opposing oxidative and nitrosative stress. However, in neurological disorders such as PD, low levels of SOD in erythrocytes have been observed [44]. Moreover, in the spinal cord mitochondria of ALS mice, accumulation of misfolded mutant SOD1 can occur, causing mitochondrial damage [45].

CAT is a tetrameric enzyme containing four iron–heme groups and it is mainly located in peroxisomes. It breaks down two hydrogen peroxide molecules into one molecule of oxygen and two molecules of water in a two-step reaction [46]. Catalase deficiency or malfunctioning is associated with oxidative stress and human diseases [47]. For example, β-amyloid has been observed to inhibit catalase activity, leading to an increase in oxygen peroxide. On the contrary, the addition of catalase exhibited protective effects against Aβ by the reduction of oxygen peroxide levels and, ultimately, protein and lipid peroxidation [48].

### 2.4. Heme Proteins and Iron

Amongst the molecules implicated in oxygen metabolism, heme proteins are fundamental for their role in oxygen delivery, as can be seen with the following formula:

*Oxygen delivery* = *cardiac output* × *systemic oxygen saturation* × *hemoglobin*

A biological response to hypoxia to limit the damage to the neuronal system is to increase the demand for iron, as this is a central component of hemoglobin. Hypoxia-inducible factor (HIF)-induced EPO production can increase intraerythrocytic hemoglobin and thus protect neurons from excessive hypoxia and hyperoxia [49]. During hypoxia, hemoglobin undergoes conformational changes that allow the release of oxygen to the tissues, thus increasing oxygen delivery to the cells and preventing damage due to oxygen deprivation. On the contrary, during hyperoxia, hemoglobin alters its affinity for oxygen by binding to oxygen more strongly, thus preventing excessive oxygen delivery and avoiding damage due to oxygen toxicity [50]. Even so, extracellular free hemoglobin and its degradation products (such as heme and free iron) can also lead to an excessive inflammatory immune response and oxidative stress, subsequently interacting with pathological processes in neurodegenerative disorders [51] and iron-induced apoptosis (ferroptosis).

Multiple ischemic and/or hemorrhagic events are the most known situations in which hemoglobin catabolism can play a key role in the damage. From excess heme catabolism, the released iron enhances the Fenton reaction, which increases the oxidative damage induced by hypoxia and ischemia-reperfusion damage [52].

### 2.5. Mitochondria and ROS

Mitochondria are fundamental organelles that are especially relevant for their role in the production of cell energy, ROS, control of calcium homeostasis and cell death [53,54]. These processes acquire a specific relevance in the brain, as neurons require energy production and calcium availability for synaptic transmission [53,54]. Mitochondria present their own life cycle, which comprises four stages: biogenesis, fusion, fission and degradation [54]. Specifically, an increase in the cell’s energetic demands leads to mitochondrial biogenesis. These organelles can then undergo fusion or fission in response to alterations in energy requirements [55]. Lastly, mitochondria that are found to be dysfunctional are removed though the autophagic process termed “mitophagy”, where the mitochondria are fragmented and degraded in the lysosomes [56]. In multiple neurological diseases, both gene mutations and environmental factors can influence mitochondrial biogenesis and health.

It is well known that mitochondrial ROS (mROS) are required for a plethora of physiological functions, such as signal transduction, aging, stem cell differentiation and proliferation, wound healing, hypoxic adaptation and insulin signaling [57,58]. The production and neutralization by endogenous antioxidants of mitochondrial ROS (mROS) are tightly regulated processes in order to maintain low levels of mROS [59]. Even if ROS are formed during ATP synthesis by complexes I and III of the electron transport chain [60], they can alter all the complexes of the mitochondrial respiratory chain when they are overproduced [61], generating more ROS. Consequently, the lipids of the inner mitochondrial membrane can undergo lipid peroxidation, leading to permeability of the inner mitochondrial membrane [62]. Moreover, since mitochondrial DNA lacks histones, it tends to be more prone to mutations mediated by ROS [63,64].

### 2.6. Protein Misfolding

Protein folding has been defined as a set of physical processes by which a newly synthesized polypeptide sequence transforms itself into a highly organized three-dimensional structure, which represents, among all possible conformations, the most stable with the lowest free-energy [65]. This mechanism depends on the amino-acid sequence intrinsic properties, but it is also due to influences from the cellular environment (e.g., ribosomal protein synthesis, interactions with other proteins or nucleic acids and chaperone-mediating folding) [66]. An increasing number of evidence has demonstrated that the main events in many neurodegenerative diseases include the misfolding, oligomerization and accumulation of cerebral proteins. This process usually leads to cellular dysfunction, loss of synaptic connections and brain damage [67]. Protein misfolding and aggregation seem to be remarkably similar steps in most neurological disorders, even if the multiple disease-associated proteins do not exhibit complementarity in terms of sequence, size, structure or function [68].

The idea that neurotoxicity exclusively depends on insoluble fibrils is still under investigation [69]. Indeed, they may represent a mechanism to escape and limit the pre-fibrillar oligomeric intermediates formation [69]. In Alzheimer’s disease (AD), the main constituents of amyloid plaques are βA (1–40) and βA (1–42) peptides, which are deposited extracellularly, whilst neurofibrillary tangles (NFTs) are intracellular aggregates [70]. In Parkinson’s disease (PD), the intrinsically disordered α-synuclein misfolds and leads to the formation of Lewy bodies, particularly in dopaminergic and noradrenergic neurons [71]. Moreover, misfolded disease-associated proteins, mainly α-syn and βA peptides, exhibit prion-like behavior [72]. In amyotrophic lateral sclerosis (ALS), several polypeptides are inclined to misfold and aggregate, such as Tar DNA binding protein-43 (TDP-43), superoxide dismutase 1 (SOD1) and ubiquilin-2 [73]. Protein aggregation in these diseases will be explained in further detail in the following section. Furthermore, as a result of the poliQ expansion, mutated huntingtin (mHtt) is prone to aggregate and accumulate [74].

In recent years, the destructive effect of oligomers and misfolded proteins has been studied in-depth in order to understand the molecular mechanisms at its base. In this context, new findings have reported that ROS and reactive nitrogen species (RNS) may play a crucial role in amino acid chains’ aggregation and protein misfolding, resulting in an another pathological trigger of NDs [75]. Particularly, these molecules can cause post-translational modification (PTMs) in proteins, leading to structural changes that lead to protein destabilization and aggregation. An example of this is represented by both S-nitrosylation and S-glutathionylation in reactive thiols by the protein disulfide isomerase [76]. S-nitrosylation consists of the addition of a nitric oxide to the thiol group of a cysteine, while S-glutathionylation is referred to a glutathione group (GSH) added at the thiol group of a cysteine [77]. These redox PTMs of cysteine residues can affect protein stability and structure, leading to an aberrant neuronal signal transduction pathway [78]. Moreover, biological processes can be affected by H_2_O_2_, which is a secondary messenger implicated in redox PMTs, capable of inducing oxidation in cysteine residues of the target protein [79].

### 2.7. Neuroinflammation

The increase in ROS production from endogenous or exogenous sources can induce an aberrant signaling inside the cells, ultimately leading to an excessive inflammatory response in the body. While in physiological conditions, the inflammatory processes represent a defense mechanism and are self-limited, in chronic oxidative stress, pro-inflammatory responses are predominant, leading to neurological dysfunctions [80]. Cellular and immune factors, such as specialized macrophages and lymphocytes, cytokines and pattern-recognition receptors, are the contributing players of neuroinflammation; these proinflammatory mediators are either released locally within the central nervous system (CNS) or engaged from the peripheral system, as a result of the destruction of the blood–brain barrier. This in turn leads to the activation of the glial cells, such as microglia and astrocytes. Therefore, considering neuroinflammation, the instantaneous focus turns to the “specialized” immune system, however, glia, mast cells, infiltrating leukocytes and ROS can lead to inflammatory responses following an injury [81].

Among the cells that participate in the inflammation of the CNS, microglia accounts for 1–16% of the total cell population in the brain [82]. Microglial cells are the main immune brain effectors, supervising their environment in forecast of an insult or damage and exerting immunosurveillance [83]. In homeostatic terms, microglial cells control and regulate both cell death and neurogenesis, contributing to synaptic maturation; when activated, they phagocytose cellular debris, present antigens to T cells and subsequently release cytokines/chemokines [84]. Microglial cells’ activation can be induced by several molecules, such as matrix metalloproteinase 3 (MMP-3), α-synuclein, amyloid beta peptide (Aβ), neuromelanin and ATP, as well as from cell damage signals and an increase of ROS [85].

Astrocytes account for 20–30% of the brain glial cells. They are crucial contributors of neuroinflammation and they have a role in a wide number of neurodegenerative diseases [86]. Physiologically, astrocytes can exert activities that are essential for neural survival, releasing several neurotrophic factors, maintaining the blood–brain barrier (BBB) integrity and regulating axonal outgrowth and myelination [87]. Injury leads to an increase in astrocyte reactivity, and they can then release chemokines, cytokines and trophic factors [88].

Lastly, the redox alterations can also impact the activation and differentiation of CD4+T cells. In the healthy brain, CD4+T cells are necessary for homeostasis, playing different roles in neuroprotection and neuronal destruction [89]. Th1 and Th17 produce pro-inflammatory cytokines, thus contributing to neuroinflammation through the secretion of pro-inflammatory cytokines and through the enhancement of microglia-mediated neurotoxicity up-regulating ROS and NO microglia release [90].

## 3. Neurodegenerative Diseases

Neurodegenerative diseases (NDs) represent a heterogeneous class of disorders typically characterized by the progressive degeneration on of the CNS or peripheral nervous system. Amongst the most studied NDs affecting the CNS, there are Alzheimer’s disease (AD), Parkinson’s disease (PD) and amyotrophic lateral sclerosis (ALS). Interestingly, most of these diseases present some common pathways, which all lead to neuronal impairment and, ultimately, cell death [91,92]. Disrupted axonal transport, loss of metabolic support from myelin sheaths and neuroinflammation, along with mitochondrial impairment [93], radical oxygen species and protein aggregation all seem to contribute to neuronal damage [94].

### 3.1. Alzheimer’s Disease

AD is an irreversible, progressive neurodegenerative disease that alters memory and thinking skills, ultimately leading to the inability to carry out simple tasks [95]. It is the most common cause of dementia and typically occurs in patients over 60 years of age [95]. The main pathological hallmarks of AD include the abnormal extracellular accumulation of beta-amyloid peptide (Aβ42), the intracellular depositions of abnormally phosphorylated tau-tangles and defects in synaptic function; this all leads to increased neuronal death [95,96], as reported in Table 1.

Chronic hypoxia was found to affect multiple pathological aspects of AD, such as amyloid β metabolism, tau phosphorylation, autophagy, neuroinflammation, oxidative stress, endoplasmic reticulum stress and mitochondrial and synaptic dysfunction. All of this can lead to the neurodegenerative process [97]. In response to hypoxia, the HIF-1α pathway plays a neuroprotective role, limiting this damage through the activation of neuroprotective pathways, such as the ones activated by EPO [1,11]. Moreover, a decrease in hemoglobin transcription (for example, due to clinically silent mutations) was theorized to influence intraerythrocytic and neural hemoglobin, and reduce oxygen, carbon monoxide and nitric oxide transport, all of which are aspects that could be involved in the pathogenesis of AD [50].

Oxidative stress has also been found to be implicated in the pathogenesis of AD and, indeed, ROS production can be related to Aβ plaques. Methionine 35 of the Aβ peptide could be strictly linked to oxidative stress and methionine often serves as a shield against oxidation for enzymes’ active sites [118,119]. Peptide–methionine sulfoxide reductase activity appears to be decreased in AD-affected brains, leading to ROS accumulation [120]. Moreover, the oxidation of fatty acids operated by ROS accelerates tau polymerization, and this could serve as a possible link between oxidative stress and the development of fibrillar pathology in AD [99,100]. Tau polymerization also has a genetic-related mechanism, which strongly depends on SOD1 and SOD2 expression. SOD1 is a powerful and ubiquitous antioxidant enzyme expressed in human cells whose main role consists of the protection of cells from the damaging effects of superoxide radicals [121].

Another well-characterized redox-related dysfunction in AD is that concerning mitochondrial health. Alterations in these organelles in AD appear early in the disease, indicating their crucial role in the pathogenesis [104,122]. An impairment in bioenergetics in AD brains was found with the identification of deficiencies in enzymes related to bioenergetic fluxes, such as the pyruvate dehydrogenase complex, α-ketoglutarate dehydrogenase complex and Krebs cycle enzymes [104]. Moreover, in vivo studies with positron emission tomography (PET) identified a reduced consumption of oxygen in AD brains, along with a reduction in cytochrome oxidase activity [123]. Mitochondria are also necessary for synaptic functioning and they localize at this site in order to buffer calcium levels and provide energy to sustain neurotransmitter release [124]. Even so, it is unclear whether mitochondrial dysfunction is a consequence of amyloidogenic pathology or rather a primary cause for AD [107]. The first hypothesis is based on evidence detailing Aβ aggregation as the primary cause of cellular degeneration through its interaction with specific cellular compartments (including the mitochondria) [70,107]. Aβ plaques can specifically target mitochondria, reducing the activity of electron transport chain enzymes and respiratory chain functions [108]. Interestingly, Aβ administration to cells lacking mitochondrial DNA did not lead to any toxicity, suggesting mitochondria could be crucial in the mediation of amyloidogenic pathology [125]. The hypothesis of mitochondrial cascade dysfunction as the primary cause of AD pathology is based on three cornerstones: firstly, gene inheritance defines the individual’s baseline mitochondrial functions; secondly, inherited and environmental factors lead to age-associated mitochondrial changes and degeneration; and lastly, the two previous factors influence the development of AD [107,126]. According to this, mitochondrial function affects APP expression, processing and the accumulation of amyloid plaques [126].

As previously mentioned, amyloid plaques are a primary hallmark of the disease, therefore, it is easy to highlight why protein misfolding can contribute to a redox imbalance. These plaques are formed by multiple proteins, the main one being amyloid-β (Aβ) [114,115]. Aβ is a ~4-kDa protein that is processed from the amyloid precursor protein (APP) by two different aspartyl proteases, called β- and γ-secretases [127]. The γ-secretases can cleave APP at different sites, and this leads to the production of either 40 or 42 amino acid residues. The 42 amino acid long protein is typically less abundant, but it is more prone to oligomerize and form fibrils. The excessive production of this form of Aβ could be sufficient to cause early-onset AD [128]. Even so, the exact conformation of soluble Aβ is unclear and experiments are still needed to determine what conformations of Aβ aggregates are pathogenic [129]. To this end, an electron microscopy analysis of a postmortem brain was performed, and the results show that all forms of amyloid plaques can be linked to the neuropathology [130]. Interestingly, both Aβ40 and Aβ42 are present in the fibrillar deposits of neuritic amyloid plaques, whilst diffuse plaques are not considered fibrillar and are mainly composed of Aβ42 [131]. Multiple studies demonstrated that abnormal Aβ accumulation can trigger tau pathology through the formation of neurofibrillary tangles [132]. Tau protein is encoded by the MAPT gene, localized on chromosome 17, and it is typically produced as a hydrophilic protein that presents large natively unfolded regions. It is especially enriched in the axons of developing and mature neurons [133,134]. Tau protein can be affected by an alternative splicing process that involves the N-terminal projection region and microtubule-binding domain (MBD), and this leads to the production of 4-repeat (4R) and 3-repeat (3R) tau. 3R tau expression is predominantly produced during brain development, whilst the expression of these two isoforms is strictly balanced with a 1:1 ratio in the adult brain [135]. Interestingly, maintenance of this ratio is fundamental and alterations in the 3R and 4R ratio have been implicated in AD, even if the results are conflicting and certain tau tangles-containing brain areas show increased 4R tau protein in some cases [136,137] and 3R tau in others [138]. Tau phosphorylation has also been proposed as the limiting factor in Aβ-induced neurotoxicity, as loss of either 1 or 2 tau genes protected hybrids against learning and memory deficits and excitotoxicity, which were contrary to those present in hAPPJ20 parental mice strains [139].

Lastly, neuroinflammation has been shown to play a key role in the pathogenesis of AD, mainly through microglia activation [109]. Indeed, these activated cells can interact with amyloid peptides and tau species, influencing disease progression [109]. Recent studies have focused on the potential of microglia targeting in AD, as these could represent an innovative approach to modulate disease progression [140].

### 3.2. Parkinson’s Disease

PD is the second most common neurodegenerative disease, with a progressive pathology ultimately leading to motor dysfunction defined as involuntary shaking, weakness and an altered posture [141]. It is characterized by two main hallmarks, which are Lewy bodies (LB), composed of α-synuclein (α-syn), and dopaminergic neuronal loss in the substantia nigra pars compacta (SNpc) [142]. Even so, along with these two main processes, dysfunction is also noted in mitochondrial health, oxidative balance, RNA biology and even synaptic functioning and calcium metabolism [142,143,144,145,146] (Table 1).

Amongst the processes concerning redox signaling related to PD pathogenesis, the hypoxia/HIF-1α signaling pathway was linked to the disease through gene mutations, risk factors, mitochondrial dysfunction, oxidative stress and metabolism impairment [147]. HIF-1 can impact the expression of LRRK2, and hypoxia can induce beta-synuclein accumulation [98]. Moreover, the cytokine EPO is neuroprotective in the disease [11,110,148,149,150,151,152].

Excess oxygen levels can also worsen the development of the disease, with an increase in oxidized lipids, proteins and DNA being present in PD, along with a reduction of glutathione in the SN [153]. Early-stage PD patients present a strong implication of oxidative stress, suggesting this is a crucial aspect even before the insurgence of neuronal loss [154]. ROS can be produced through numerous pathways, including activation of NADPH oxidase (NOX), cytochrome P-450 oxidase, inflammatory responses and hydrogen peroxide (H_2_O_2_) breakdown. NOX could indeed be a major player in the connection between ROS formation and PD pathogenesis, as its function and activity was found impaired in the dopaminergic neurons of PD patients, with NOX1 being increased in PD patients leading to ROS accumulation [101,155]. Interestingly, NOX2-deficient mice demonstrated protected from the neurotoxic treatment of 1-methyl-4-phenyl-1,2,5,6-tetrahydropyridine (MPTP) [156]. Amongst the other proteins involved in oxidative stress in PD, DJ-1 acts as a chaperon protein for redox changes, as well as a marker and sensor for oxidative stress [157]. LRRK2 mutations in neurons also lead to higher vulnerability to toxicity induced by ROS [158]. Indeed, LRRK2 protein was found to increase ROS generation and causes enhanced neurotoxicity [159]. Another crucial player is the transcription factor nuclear factor erythroid 2–related factor 2 (Nrf2), known as the main protein that defends against oxidative stress [160]. This transcription factor acts by binding the promoter at the antioxidant response element (ARE) in neuroprotective genes, which include antioxidant enzymes, such as heme oxygenase-1 (HO-1), glutathione cysteine ligase regulatory subunit (GCLC) and glutathione cysteine ligase modulatory subunit (GLCM) [161]. Ultimately, evidence shows that the activation of Nrf2 leads to a decrease in neurodegenerative events, whereas its inhibition exacerbates it [162].

As neural cells have been found to contain hemoglobin, it was found that heme metabolism also plays a role in PD, and this was due to an increase in hemoglobin concentration in neurons of the SN [163]. Even so, the role of heme in the pathogenesis of the disease requires further elucidation, and the degeneration of dopamine neurons in the SNpc in PD is associated with siderotic foci [164]. While some published studies revealed no relation between new PD onset and hemoglobin concentrations [165], the severity of the disease correlates with both iron metabolism and low hemoglobin [103].

PD is one of the NDs with the most implication for mitochondria’s health. The involvement of this organelle in PD was first proved in 1983 after drug users self-injected an intravenous solution of MPTP, an inhibitor of mitochondrial chain complex I, which led to the development of parkinsonism [166]. Toxins such as MPTP and rotenone impair the mitochondrial electron transport complex and hinder mitochondrial movement, whilst they also lead to an increase in the mitochondrial permeability transition, ROS generation and nitric oxide synthase activity. The complex I activity results demonstrated impairment not only in the SN, but also in the skeletal muscles, platelets and leukocytes of PD patients [107]. Mitochondrial dysfunctions are present in both sporadic and familial PD, and they can occur from early on in PD pathogenesis [167]. Common PD-causing genes, such as Parkin, DJ, PINK1, α-SYN and LRRK2, can have pathogenic mutations that directly or indirectly support that mitochondrial dysfunction is implicated in familial PD [167]. PINK1 and Parkin are crucial in this process, and when PINK1 is activated, it recruits Parkin, resulting in the ubiquitination of the outer mitochondrial membrane proteins, which leads to mitophagy [107]. Moreover, α-syn can impact the mitochondrial protein import machinery and affect complex I activity, DJ-1 can protect neurons against oxidative stress, and LRRK2 can potentiate the pro-fission activity of DRP1 [53,94]. Mitochondrial dysfunction is also relevant for sporadic forms of PD, as evidence obtained in fibroblasts and peripheral blood mononuclear cells (PBMCs) from PD patients identified mitochondrial and lysosomal dysfunctions [105,106]. Interestingly, mitochondria could be crucial regulators of the selective dopaminergic neuronal susceptibility in the SNpc [167]. Indeed, SNpc neurons present an extensive axonal harbor, which leads to a high bioenergetic demand [168]. Moreover, SNpc neurons appear to undergo an increased rate of mitophagy as opposed to dopaminergic neurons present in other areas, such as the ventral tegmental area [115]. High fluxes of cytosolic calcium during neuronal activity are also present in dopaminergic neurons of the SNpc, and modulation of calcium channel CaV1 appears to decrease mitochondrial oxidative stress without altering pacemaker activity [115].

The study of protein misfolding and aggregation in PD has become crucial in the disease, as mutations in the gene encoding for α-syn (SNCA) cause familial forms of the disease, and moreover, the protein is a main component of LB, a key driver in PD pathology [116]. α-syn is a member of the synuclein family and is composed of three proteins (including β- and γ-synucleins) all encoded by three different genes. In humans, the predominant cerebral form of α-syn is a small, soluble protein of 140 residues, representing about 1% of the total neuronal proteins, where it localizes in presynaptic nerve terminals. α-syn is composed of three domains: an N-terminal region (composed of an 11–amino acid sequence repeat resembling the apolipoproteins α-helical lipid-binding motif), a central region composed of hydrophobic amino acids (that tends to favor aggregation and, ultimately, amyloid fibrils formation) and a hydrophilic C-terminal region rich in proline, glutamate and aspartate, which could confer chaperone activity to α-syn [169]. Furthermore, LB are characterized by phosphorylated, nitrated and oxidized α-syn, and dysregulated post-translational modifications of α-syn may lead to the formation of pathological inclusions [170]. Genetic evidence also implicates several other proteins related to PD pathogenesis in protein misfolding mechanisms, some of which have been found to be associated with mitochondria [171]. One of these is the enzyme parkin, a ubiquitin E3-ligase encoded by the PARK2 locus. Impaired parkin could lead to a toxic accumulation of its substrates, and missense mutations can impact protein solubility, leading to its aggregation in aggresome-like inclusions [172]. Parkin loss of solubility is also linked to α-syn accumulation/aggregation, but the precise mechanism is yet to be characterized [173]. The development of all these redox-related phenomenon can also exacerbate neuroinflammation, as this has been reported to act in dopaminergic neural cell death in PD. Indeed, postmortem SN from human PD brains reveal deep activation of microglia and lymphocytes infiltration when compared to controls [110,111,112].

### 3.3. Amyotrophic Lateral Sclerosis

ALS is a neurodegenerative disease characterized by upper and lower motor neuron loss in the spinal cord, brainstem and motor cortex [174]. The disease presents with motor symptoms that include muscular atrophy and spasticity, leading to paralysis within 3–5 years of onset, with the primary cause of death being respiratory failure [175]. ALS is a complex disease and most of the molecular pathways leading to neuronal degeneration are yet to be characterized [113,175,176,177,178,179]. Disrupted axonal transport, loss of metabolic support from myelin sheaths and neuroinflammation, along with mitochondrial impairment, ROS formation and protein aggregation all seem to contribute to motor neuronal damage [180,181], as summarized in Table 1.

Multiple studies have inferred the role of ROS in ALS, the main evidence being that there is increased oxidative stress in ALS postmortem tissue with respect to control samples [182]. Lipid oxidation markers were also found in spinal cords from sALS patients, whilst they were undetected in controls [102]. 8-hydroxy-2′-deoxyguanosine (8-OHdG), a marker of oxidized DNA, presented an increased abundance in ALS patients’ whole cervical spinal cord [183]. Oxidative stress markers were also analyzed in the cerebrospinal fluid (CSF) of early-stage ALS patients; these markers include increased levels of 4-hydroxynonenal (for lipid peroxidation) and ascorbate free radicals [184,185]. Specifically, D90A, A4V and G93A mutations in the SOD1 gene impact SOD1 activity, resulting in the increase of highly toxic hydroxyl radicals, which lead to neurotoxicity and, consequently, neuronal death [100]. It is widely accepted that mutant SOD1 toxicity is the result of an unknown gain of function [186]. When investigating the impact of mutant SOD1 expression in motor neuronal cells with a microarray approach, the results highlighted the downregulation of genes involved in the antioxidant response, such as transcription factor Nrf2, members of the glutathione S-transferase family and peroxiredoxins [187].

Moreover, in ALS, evidence shows that axonal mitochondria have peculiar morphological features, which consist of rounding up of the mitochondria, networks fragmentation and swelling of internal cristae [188,189]. Moreover, high intracellular calcium concentration in degenerating motor neurons interferes with mitochondrial transport, causing fragmentation or morphological changes, bioenergetics dysfunctions and mitochondrial apoptosis [189,190]. Mitochondria autophagy, known as mitophagy, is impaired in ALS, leading to the irreversible degradation of mitochondria. Physiologically, the autophagy receptor OPTN and its kinase TBK1 are responsible for autophagosome engulfment of damaged mitochondria [191]. Mutations in both OPTN and TBK1 have been linked to ALS, and a loss of function of these genes leads to impaired mitophagy and accumulation of damaged mitochondria [191,192]. In both sALS and fALS patients, the impairment of axonal transport is one of the earliest pathological events in the pathogenesis of the disease [193]. Interestingly, in murine models harboring the SOD1G85R and SOD1G37R mutations, the number of axonal mitochondria is reduced and their distribution is inhomogeneous throughout the axon [194].

ALS also presents, as a main hallmark, cytoplasmic inclusions or aggregates in neuronal cells and oligodendrocytes. Although the spinal cord is the main affected area of the pathology, these inclusions are also found in the brain regions, such as the frontal and temporal cortices, hippocampus and cerebellum [113]. ALS inclusions can be defined as hyaline- or skein-like and they are organized as filaments with a random orientation and covered by fine granules [195]. Another subclass of ALS inclusions is that of Bunina bodies, small ubiquitin-negative inclusions [196]. The main protein present in these aggregates is TDP-43, a DNA- and RNA-binding protein that can impact nuclear RNA metabolism through an alteration of splicing, transcriptional repression, miRNA synthesis, mRNA nucleo-cytoplasmic shuttling and RNA transport [197,198]. As TDP-43 exerts a nuclear function, its localization in healthy neurons is in the nucleus, but ALS pathology leads to a cytoplasmic aggregation of TDP43 and a decrease in its nuclear levels [199]. The TDP43-encoding gene, TARDBP, is mutated in 1–2% of fALS and sALS cases, with mutations being localized in the region that encodes for the C-terminal glycine-rich domain of the protein. Interestingly, this domain is implicated in the splicing activity of TDP43 and its ability to interact with other proteins [200]. Furthermore, in ALS patients, the protein is cleaved in hyperphosphorylated C-terminal fragments of 18–26 and 35 kDa, which are aggregation-prone and toxic [201]. fALS-causing mutations were also found in the RNA-binding protein FUS [202]. In contrast to TDP-43, mutant FUS protein does not present post-translational modifications, but it is enriched in the insoluble fraction in the pathology [203]. FUS is also implicated in processes that involve nucleic acids’ metabolism, such as DNA repair, transcription, splicing and miRNA processing [197]. Moreover, it is responsible for shuttling mRNA from the nucleus to the cytoplasm, and most ALS-linked FUS mutations reported are localized in the nuclear localization sequence (NLS) of the protein, which ultimately results in the inability of FUS to shuttle back to the nucleus [204,205]. Both FUS and TDP-43 contain prion-like domains enriched in asparagine, glutamine, tyrosine and glycine residues. These regions can be present in two states: an unfolded and an aggregated state, and the aggregation of FUS and TDP-43 appears to be due to alterations in these domains [206,207]. The RNA-binding properties of FUS and TDP-43 are also responsible for the proteins’ toxicity, as this activity is essential to create RNA–protein complexes, such as stress granules [208].

The Immune system is also involved in ALS, as it stimulates the production of pro-inflammatory cytokines, such as IFN-γ and TNFα, expressing cyclooxygenase-2 (COX-2), which can be found in CSF, serum and urine samples of both types of ALS patients [117]. 

## 4. Pediatric Neurological Disorders

Oxidative damage related to an oxygen imbalance can be considered a hallmark of many adult-onset neurodegenerative diseases, including AD, PD and ALS. However, this condition is also observed in many pediatric onset neurodegenerative disorders, including genetic white matter disorders, such as adrenoleukodystrophy (X-ALD) and Pelizaeus–Merzbacher disease (PMD); primary neuronal disorders, such as spinal muscular atrophy (SMA); and neurometabolic conditions, such as mucopolysaccharidoses (MPS) and Pelizaeus–Merzbacher disease (PMD) [209,210,211].

### 4.1. Adrenoleukodystrophy

**Figure 2:**
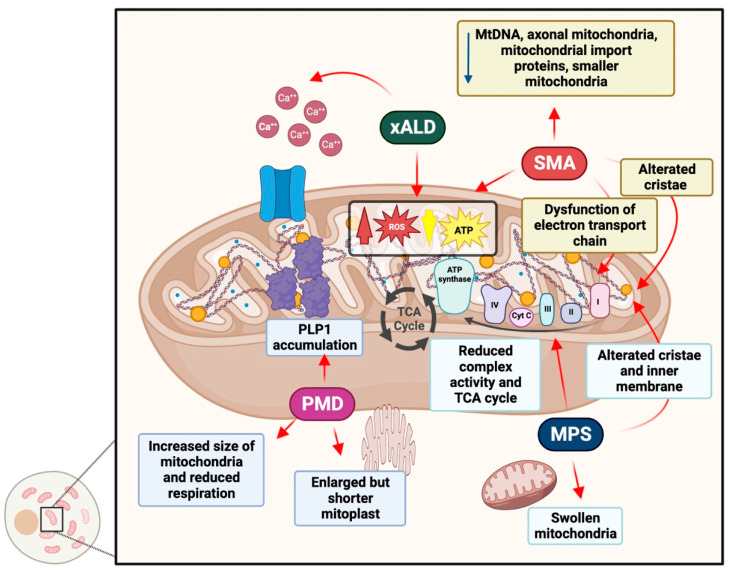
Schematic representation of mitochondrial damage mechanisms in some examples of pediatric-onset neurodegenerative diseases.

X-ALD is a genetic disorder that mostly follows an X-linked inheritance pattern, and it is the most common peroxisomal disorder that can affect both males and females. It presents an estimated birth incidence of about 1/14,700 [212,213]. This disorder is caused by mutations in the ABCD1 gene, encoding for a member of the superfamily of ATP-binding cassette (ABC), transporters also known as ALD [214]. Specifically, ALDP is implicated in the transport of straight-chain very long-chain fatty acid (VLCFA) as CoA esters from the cytosol to the peroxisome lumen, where they are metabolized via β-oxidation [215]. Defects in the activity of the ABCD1 transporter lead to VLCFA excess and the subsequent accumulation in many areas, especially in the brain, spinal cord and adrenal cortex [216]. Clinical presentation of X-ALD phenotypes depends on a progressive central demyelination in the brain and/or slowly progressing axonal degeneration resulting in cognitive decline, spastic quadriparesis and visual and hearing impairment [217]. The exact molecular pathway involved in X-ALD pathogenesis is only partially known, but many contributors of this disease have been identified. These include oxidative stress and mitochondrial dysfunction [218]. Indeed, excess VLCFA induces oxidative stress acting on the unfolded protein response (UPR), a cellular mechanism necessary to maintain oxygen homeostasis [219]. This process is highly regulated by an ER-located transmembrane receptor called protein kinase RNA-like endoplasmic reticulum kinase (PERK), as it has recently emerged that an increase of VLCFA, especially C26:0, induces ER stress in human ALD fibroblasts with a subsequent activation of the PERK pathway [219]. PERK activation leads to the increase of ROS generation and to the decrease of mitochondrial membrane potential [218]. Recent research has demonstrated that this condition is a major contributor of X-ALD pathogenesis in the brain, fibroblasts, erythrocytes and peripheral mononuclear cells (PBMCs) of x-ALD patients [218,220]. Specifically, once produced, mitochondrial ROS may oxidize and inhibit the oxidative phosphorylation system (OXPHOS), inducing a decline in ATP production [221]. ROS promote the opening of the mitochondrial permeability transition pore (mtPTP), leading to a massive efflux of calcium from the mitochondria to the cytosol (Figure 2). As a consequence, calcium overload in the brain leads to the activation of calpains, which dismantle the microtubule structure [222]. In the brain, all these events impair the correct axonal transport along the axon with a loss of axonal continuity and, eventually, axonal disruption [223]. In X-ALD, there is an unbalanced ROS/ATP/Ca2+ homeostasis, but this mechanism has not been fully characterized yet. However, it is hypothesized that excess in VLCFA, especially C26:0, could alter the permeability of the inner mitochondrial membrane through the increase of the membrane microviscosity, provoking its disruption [224].

### 4.2. Pelizaeus–Merzbacher Disease

Pelizaeus–Merzbacher disease (PMD; OMIM #312080) is an X-linked recessive hypomyelinating leukodystrophy [225]. PMD is caused by hemizygous mutations in males or heterozygous variants in females with skewed X inactivation in the PLP1 gene, which encodes for both proteolipid protein-1 and the alternative splicing variant DM20, two of the most abundant proteins in the CNS myelin [226,227]. According to the age of onset of symptoms, PMD can be classified as connatal, transitional, classical or intermediate [228]. In any case, when the myelination process fails, axonal damage and dysfunction arise, with deleterious consequences on cognition deficit and motor abilities [229]. From the genetic point of view, PLP1 can be altered in several ways: it can be subjected to gene duplication (triplication or even quintuplication in rare cases), deletions, point mutations or null mutations [228,230]. These mutations reflect different pathological molecular mechanisms and correlate with a wide variety of clinical phenotypes [228]. For example, point mutations, known to cause mutant protein misfolding, lead to PLP1 accumulation in ER, ER stress and, ultimately, cellular toxicity. Differently, PLP1 duplication, associated with a more severe phenotype, is characterized by an abnormal accumulation of PLP1, which fatally affects myelinating oligodendrocytes. Recent evidence suggests that mitochondria are involved in the promotion of cellular stress and damage [227,231]. In fact, in the PLP-tg66/66 brain, the mouse model that recapitulates the duplication PMD phenotype, a reduction in ATP levels and mitochondrial membrane potential, along with an increase in cytochrome c oxidase, have been reported [231] (Figure 2). In addition, besides an increase in number and size of the mitochondria in oligodendrocytes and axons [231], PLP1 has been observed to co-localize in the mitochondrial membrane, destabilizing extracellular pH [232,233]. Moreover, in both the PLP-tg66/66 spinal cord and in human PMD fibroblasts, Ruiz and collaborators observed increased free radical generation and oxidative damage, with a concomitant decrease in antioxidant defenses, such as glutathione, while the mitochondrial dysfunction was prevented by the administration of the antioxidant NAC [231].

### 4.3. Spinal Muscular Atrophy

SMA is a neuromuscular disorder characterized by progressive degeneration of the motor neurons of the anterior horns of the spinal cord, which results in muscle weakness and atrophy [234]. This pathology is caused by biallelic deletions or mutations in the survival motor neuron 1 gene (*SMN1*; 5q13.2), leading to the deficiency of the SMN protein [235]. Due to an inverted duplication on chromosome 5q13, the human genome contains an almost identical gene, *SMN2*, that mainly produces a transcript lacking exon 7, resulting in production of a truncated, less stable SMN protein (85–90%). Approximately 10–15% of the full-length protein is encoded by the *SMN2* gene, thus generating a very small amount of functional SMN protein [236,237]. SMN is a ubiquitous RNA-binding protein, which is involved in collaboration with partner proteins, such as Gemin2–8 [238], in the biogenesis of spliceosomal small nuclear ribonucleoproteins, snRNPs, (which are fundamental for pre-mRNA splicing) and trafficking of mRNAs to axon terminals [239].

Recent evidence suggests that SMA pathology is linked to a decrease in mitochondrial respiration and in the activity of oxidative phosphorylation enzymes, and a concomitant increase in ROS levels [240,241]. This energetic impairment has been observed in various animal and cell culture models [241,242,243]. Specifically, the proteome analysis of motor neurons isolated from an SMA mouse model showed disturbed energy homeostasis due to dysfunction of the electron transport chain in the mitochondrial complex I [244]. This resulted in a higher ROS production and lower basal ATP levels that cause an increased protein carbonylation and impaired mRNA translation at the initiation step, contributing to reduced protein synthesis efficiency in SMA motor neurons [244]. Interestingly, supplementation of pyruvate led to an increase in SMN protein levels by reduction of ROS via the mTOR pathway [244].

Moreover, morphological abnormalities of mitochondria, including alterations in cristae, were found in animal models of SMA where smaller mitochondria were linked to reduced SMN protein levels [245,246]. Additionally, the number and density of axonal mitochondria are reduced in murine [247,248] and in vitro [246] models of SMA compared to wild-type ones (Figure 2). Reduced mitochondrial DNA (mtDNA) was also detected in patient muscle tissue [249], along with a decreased expression of mitochondrial import proteins found in patient-derived spinal motor neurons [250] and patient muscle biopsies [249]. In this context, a protein involved in synaptic transmission and already implicated in the SMN pathway, stasimon, interacts with VDAC1, an outer mitochondrial membrane import protein. VDAC1–stasimon interactions may be affected by reduced SMN levels, implicating a mitochondrial protein import in the SMA pathology [251,252]. Interestingly, the typical elongated mitochondrial network may also be reduced in SMA models [246]. This is supported by patient-derived cell culture models of SMA showing reduced mitochondrial trafficking due to the dissociation of SMN interactions between ARX-2 and actin filaments [210,246].

Studies have also reported an alteration in apoptosis regulatory proteins in patients and models of SMA [253]. SMN protein interacts with Bcl2 (the anti-apoptotic and outer mitochondrial membrane protein) to exert a synergistic effect against apoptosis [254]. There is evidence implicating Bcl-2 family members in SMA in cell culture models, animal models and patient tissue [255,256,257]. Another SMN interactor is p53, a transcription factor involved in cell stress that can both induce apoptosis (nuclear p53) and repress autophagy (cytosolic p53). In a mouse model of SMA, nuclear p53 activity was upregulated [258], while the SMN/p53 interaction correlates with SMA disease severity in patient-derived fibroblasts [259]. Finally, another apoptosis-inducing protein is ZPR1, which may impact disease severity [260].

Overall, these changes indicate mitochondrial impairment in SMA disease.

Evidence for neuroinflammation in SMA pathogenesis remains elusive, despite its likely contribution to disease onset and/or progression. Increased astrogliosis was observed in autopsies of patients and in a murine model, while microglia and T-cells have never been thoroughly investigated [261,262,263]. Studies have shown abnormalities in immune organs (e.g., spleen) and T-cell alterations in SMA mouse models, resulting in an abnormal neuroinflammatory response and exacerbation of the disease [264]. Importantly, functional studies investigating the status of T-cells and their protective or cytotoxic function in SMA may provide us insight on neuroinflammation in the pathogenesis of SMA.

### 4.4. Mucopolysaccharidoses

Mucopolysaccharidoses (MPS) are a group of inherited disorders belonging to the lysosomal storage diseases. Eleven subtypes of MPS are currently known, distinguishable for the defective or absent enzyme involved in the abnormal lysosomal accumulation of glycosaminoglycans (GAGs) [265]. Physiologically, GAGs, present in every mammalian tissue [266], play a pivotal role not only in structural scaffolding, but also in several metabolic pathways: regulation of cell growth, proliferation, cell adhesion, anticoagulation and wound repair [265]. However, even if the pathophysiology of MPS has not been fully clarified, inflammation, oxidative stress and mitochondrial dysfunction contribute to the disease progression in a cross-linked cascade of events.

It has been reported that a GAG structure, mimicking that of lipopolysaccharide (LPS), was able to activate Toll-like receptor 4 (TLR-4), promoting the secretion of pro-inflammatory cytokines in an animal model of MPS [267]. GAG storage leads to abnormal morphology in lysosomes, leading to lysosomal swelling and vacuolation, as reported in MPS mice neurons and glia, as well as in MPS human neural stem cells [268]. Along with altered lysosomal homeostasis, another important source of ROS in MPS diseases is caused by mitochondrial dysfunction [211]. In particular, in MPSIIIC mice brains, an increased number of swollen mitochondria was observed 5 months after birth. These mitochondria were characterized by morphological aberrations, such as the disorganization of the cristae in the inner membrane [269]. In addition, mitochondrial respiration has also been reported to be impaired in the MPSIIIC mouse model. In fact, the activity of complexes II, III and IV and citrate synthase was reduced [269] (Figure 2). Mitochondrial-mediated oxidative stress has been associated with the worsening of the inflammatory process through the release of ROS and RNS in MPS. The NADPH-oxidase complex, specifically expressed by microglia, increases in the MPSIIIB mouse model, inducing macrophage inflammatory protein-1a and caspase 11, suggesting that neuroinflammation could generate oxidative stress, leading to apoptosis [270]. Similarly, elevated levels of TNF-a were observed in MPSI, II and III patients [271]. A global impairment in the redox status has also been observed in MPS patients: an increase in plasmatic lipid peroxidation and protein oxidation, as well as alterations in erythrocyte SOD and CAT activities have been reported for MPSI, II and IIIB patients [272,273].

Besides GAG accumulation, secondary storage of substances such as glycosphingolipids, phospholipids and cholesterol has been documented [274]. Generally, the abnormal accumulation of metabolites that become toxic in cells and tissues is considered the main cause of the increased generation of ROS [275]. In both MPS animal models and patients’ brains, histological analyses revealed that an impaired accumulation of GM2 and GM3 gangliosides occurred [276,277]. Secondary lipid accumulation has also been detected by Nile red staining in stem cells-iPSC derived from MPSIIIB fibroblasts [278]. The storage of gangliosides in the CNS has been linked to compromised neuronal function and survival, preventing calcium uptake within the endoplasmic reticulum [279]. In addition, the sequestration of cholesterol in neurons and glia observed in the MPSIIIA mouse model was associated with an impairment of endosomal transport [280]. It has been demonstrated that abnormal trafficking also has an impact on the autophagy–lysosomal pathway (ALP), the process by which intracellular macromolecules or damaged organelles are degraded [281]. ALP also plays a pivotal role in protein homeostasis and in the removal of protein aggregates. Mice lacking ALP components showed neuronal accumulation of aggregate-prone proteins, leading to neurodegeneration and oxidative stress [282]. Thus, consequent to the degeneration of the autophagy–lysosomal pathway (as described above for PD), insoluble aggregation of α-syn occurred in neurons of a mouse model of MPSIIIA [283]. Monaco and collaborators reported that α-syn gradually accumulates together with other amyloid proteins, including prion protein, phosphorylated tau and Ab, mostly into the lysosomes of neuronal cell bodies in MPSIIIA and MPSIIIC mice [284]. α-syn was also found to be accumulated in several brain regions of human MPSIIIB patients [285] and in the cerebral cortex of post-mortem tissues of MPSIIIA patients [286].

## 5. Therapeutic Prospects

Oxygen metabolism is an essential biological step for every vertebrate organism and for this reason, as previously discussed, oxidative stress and processes affected by an oxygen imbalance are the main features of many NDs and PNDs. Indeed, pathological conditions in the brain have been related with increased oxidative stress, which leads to redox imbalance and inflammation [287]. Researchers have tried for years to develop a new strategy to prevent or cure oxidative stress, but only few successes have been reported.

### 5.1. Antioxidants

Antioxidants are exogenous or endogenous molecules that can neutralize ROS and other kinds of free radicals. These molecules are contained in numerous foods, such as flavonoids and phenolic compounds, lipoic acid (thioctic acid), ubiquinone and idebenone, β-carotene and vitamin C [288]. The typical mechanism of antioxidants’ action involves direct and indirect pathways. These include scavenging and metal chelating effects, mimicking or upregulating antioxidant enzymes, activation of Nrf2, increasing the activity of sirtuins and inhibition of pro-oxidant enzymes, among others. Recent findings refer to polyphenolics, thiolics and SOD mimetics not only as inducers of heme oxygenase-1 and nitric oxide synthase, but also as activators of Nrf2 and NADPH oxidase inhibitors [288].

These molecules have been widely studied in neurological disorders and have been found to give promising results in animal models of AD [289,290]. Amongst them, vitamin E (alpha tocopherol) acts as an antioxidant molecule partially restoring cognitive functions in individuals with AD [291,292]. Inconclusive results were also obtained in clinical trials with curcumin, which is a polyphenolic compound with antioxidant and anti-inflammatory effects [293]. The compounds that directly target mitochondria include coenzyme Q10, idebenone, creatine, latrepirdine, methylene blue, triterpenoids, curcumin, Ginkgo biloba extract and omega-3 polyunsaturated fatty acids [288]. Multiple studies have tested these compounds using both in vivo and in vitro models of AD, demonstrating their effectiveness in protecting the brain against Aβ-induced oxidative stress, synaptic loss, mitochondrial dysfunction and abnormal calcium homeostasis [294]. Moreover, curcuminoids have been found to attenuate SMA-related processes in patient-derived fibroblasts [295], and, indeed, the use of these molecules has been proposed as therapy for the disease [296]. Antioxidant molecules (specifically N-acetyl-cysteine, α-lipoic acid and α-tocopherol) were also found to attenuate symptoms in a murine model of X-adrenoleukodystrophy [222], and this combination was also used in a phase-II pilot study, with positive outcomes [297].

Among the antioxidants, GSH is the most abundant; its nuclear accumulation plays an important role in cell proliferation, oxidative signaling and in the control of the redox state of critical protein sulfhydryls, which are necessary for DNA repair and expression [298]. In the brain, GSH levels decrease with age, which could impact cognitive function. Moreover, a decrease in GSH levels is associated with microglial activation and endothelial dysfunction, both of which can contribute to the impairment of brain function [298]. Since GSH is implicated in different pathways related to AD, different strategies to improve plasma and brain availability have been studied to restore intracellular levels and prevent the alterations observed in AD. GSH synthesis is often restricted by the availability of L-cysteine, which can be obtained by the breakdown of GSH or from proteins, synthesized endogenously, or from diet. As a result, several approaches were explored, including investigating the correlation between diet and GSH tissue levels [299], developing pharmacological L-cysteine prodrugs [300] and administering γ-GC orally [301]. However, to date, clinical trials did not highlight significant restoration of GSH levels [302]. Recently, supplementation with γ-GCS for 3 months was found to be associated with a reduction of brain oxidative stress, neuroinflammation and maintenance of the antioxidant status in an AD mouse model [303]. Thus, given the important role of GSH in biological activities, new pharmacological approaches to increase or to maintain its levels should be addressed. To date, there is only one clinical trial (start: February 2021, end: May 2025) that aims to restore GSH levels and examine the effects on cognitive improvement (NCT04740580) [304]. Specifically, since it has been observed that the depletion of GSH can be restored by using glycine and cysteine (provided as N-acetylcysteine NAC), this early phase 1 trial will compare the effects of 24 weeks of GlyNAC supplementation with respect to an alanine placebo in patients with AD. The trial aims to assess changes in cognition, GSH levels, oxidative stress, brain glucose uptake, brain inflammation and insulin resistance (NCT04740580) [304].

### 5.2. Mitochondria-Targeting Drugs

Recently, mitochondrial malfunction and oxidative damage have gained much more attention, as significant evidence suggests their role in the pathogenic mechanism of NDs and new possible targets of novel treatments for these disorders [305]. For these reasons, the cytokine EPO has also been studied as a neuroprotective potential therapy, as its effects were studied in numerous in vitro and in vivo ND models and in two clinical trials [152,306]. Several other molecules display mitochondrial protective activity, such as analogues of SOD-CAT, which present beneficial effects in various models of oxidative stress. For example, silent Mn complexes were effective when directly testing mammalian mitochondrial oxidative stress in SOD2 KO mice [307] and they were found to have protective effects against oxidative stress in animal models of several ND, such as PD [308], ALS [309], AD [310] and stroke [311].

Among the new therapeutic approaches, mitochondrial proteins, such as sirtuins, seem to be favorable drug targets, as they actively regulate cell survival during various physiological and pathological conditions [312]. Specifically, sirtuin 3 (SIRT3) is a mitochondrial NAD+-dependent protein deacetylase encoded by the nuclear genome, which is most commonly expressed in tissues with high oxidative capacity, such as the brain. This protein is involved in the regulation of all the complexes of the mitochondrial respiratory chain (MRC), thus modulating the typical mitochondrial bioenergetic failure implicated in PD [313]. Upregulation of SIRT3 confers neuroprotective effects in PD and other NDs [314]. Interestingly, activation of SIRT3 by icariin (ICA), a natural flavonoid glucoside isolated from the herb Epimedium grandiflorum, was found to be neuroprotective against dopaminergic neuronal cell loss in the rotenone-induced PD rat and cell models [315,316]. Several natural products are capable of stimulating SIRT3 activities. Particularly, resveratrol, a phytoalexin produced by many herbs, upregulates SIRT3 activity by activating SOD2 [317]. It has been demonstrated that resveratrol leads to dopaminergic neuroprotection activity and a decrease of oxidative stress in mitochondrial complex I-deficient PD models [318]. Honokiol is another poly-phenolic compound that modulates SIRT3 activity ameliorating motor impairment and progressive dopaminergic damage in 6-OHDA-lesioned PD mice [319]. Other natural products that modulate SIRT3 activity are kaempferol (which prevents dopaminergic neuron loss by increasing superoxide dismutase and glutathione peroxidase activities) and salidroside [320]. Given the difficulty of diffusion through the mitochondrial membranes, mitochondria-targeted compounds need to have different strategies for delivery, these being active and passive targeting [321]. In active targeting, there are interactions taking place at mitochondrial sites, such as ligand–receptor associations and antigen–antibody binding, and these can be exploited in order to take advantage of the compatibility between the physicochemical properties of the carrier molecules and those of the mitochondrial compartment. Indeed, small molecules can be efficiently targeted in mitochondria with several strategies, such as enclosure in liposomes, conjugation to lipophilic cations [321,322] and incorporation into mitochondria-targeted peptides [323].

In SMA, the molecule olesoxime, which was studies in phase III clinical trials, exerts a neuroprotective role through mitochondria targeting, with improved cellular survival in in vitro and in vivo models [210,324]. Furthermore, metformin was found to induce mitochondrial biogenesis with anti-inflammatory effects in fibroblasts obtained from patients with adrenoleukodystrophy [325].

### 5.3. Metal Protein Attenuating Compounds

Therapies aimed at targeting transition metal dysregulation are a potential treatment for specific neurological disorders. Metal protein attenuating compounds (MPACs) appear to be an emerging therapeutic approach that can lead to restoration of metal homeostasis, decreased oxidative stress and reversing or slowing disease progression. MPACs compete for binding with redox active metal ions, subsequently preventing oligomerization [326]. Many studies suggest an important role for transition metal ions in AD, enhancing Aβ aggregation and facilitating oxidative stress. Clioquinol (PBT1) is a small lipophilic compound with promising potential in the treatment of NDs due to the demonstrated ability to readily cross the BBB. Specifically, PBT1 oral administration in Tg2576 transgenic mice provoked a 49% decrease in brain Aβ burden compared with non-treated controls [327]. Moreover, the effect of the oral PBT1 treatment in a pilot phase II clinical trial of AD patients showed a statistically substantial prevention of cognitive deterioration [328]. A novel second generation MPAC PBT2 has been synthesized, characterized by a higher solubility and increased BBB permeability as compared with PBT1. The treatment with this new compound in a mouse model of AD counteracted Aβ accumulation, significantly improving cognitive performance [329]. The copper/zinc/calcium chelators, DP-109 and DP-460, were shown to be protective in ameliorating symptomatology related to ALS in a mouse transgenic model expressing G93A-mutated SOD1 [330]. Both chelators were found to improve motor activity associated with a decreased loss of lumbar spinal neurons, reduced reactive gliosis and markers of oxidative stress, as well as extend survival times of the treated mice by approximately 10% when compared with controls [331].

### 5.4. Opioids or Cannabinoids

The endocannabinoid system is fundamental in regulating myenteric neuron activity and vagal and sympathetic nerve function. For this reason, alterations in this system are found in both experimental models and post-mortem brain samples from patients with several neurological diseases [332]. Cannabinoids can primarily act by counteracting the infiltration of peripheral immune cells to the CNS, which are directly involved in the shift of the phenotypes of microglia and infiltrating macrophages from pro-inflammatory to anti-inflammatory [332]. For these anti-inflammatory effects, cannabinoids have been studied in the context of neurodegenerative diseases. In vitro and in vivo models of Aβ-induced neurotoxicity indicated that CBD can protect against Aβ-induced insults, as it reduces oxidative stress, tau phosphorylation and expression of the inducible nitric oxide synthase via the WNT–β-catenin pathway [333]. Studies performed in 6-hydroxydopamine (6-OHDA)-treated rats and lipopolysaccharide-treated rats highlighted the anti-parkinsonian effects of THC and CBD [334], probably due to the antioxidant properties of these cannabinoids. It was also demonstrated in a double-blind trial of CBD use in patients with PD that the highest dose tested (300 mg/day) improved the patients’ quality of life and reduced levodopa-induced dyskinesia in PD [335]. Regarding ALS, a selective CB2 agonist slowed disease progression in SOD1 mice, suggesting that CB2 has a protective role in ALS [336]. However, since clinical tests of cannabinoids in patients with neurological disorders are limited, and most of the mechanisms of action behind these results are still unclear [332].

### 5.5. Non-Pharmacological Interventions

Non-pharmacological treatments and lifestyle interventions, including exercise and caloric restriction, are becoming more and more relevant for their overall positive effect on health and lifespan [337]. Indeed, the Alzheimer’s Association found that regular physical exercise is a key strategy to reduce the risk of cognitive decline and the development of dementia, as regular physical activity is positively correlated with reduced oxidative stress, increased antioxidant capacity, increased anti-inflammatory effects, reduced levels of ceramides that are elevated in AD, improved Aβ clearance associated with the upregulating Aβ transporters and induced neurogenesis [338]. Even so, research is still needed to clarify the molecular mechanisms implicated in this positive effect, and a better understanding of lifestyle modifications is needed to develop efficacious therapeutic strategies for AD.

In the last few years, major advances in gene therapy research for neurological disorders have also been realized. Through the overexpression of pro-survival growth factors or targeting endogenous mutant or wild-type genes associated with disease pathogenesis, various research has highlighted the opportunity to resort to gene therapy against neurological diseases [339]. Interestingly, lentivirus-mediated expression of the lysosomal cysteine protease cathepsin B in APP transgenic mice reduced pre-existing Aβ deposits with promising results [340]. Regarding PD, it has been known that lentivirus-delivered siRNA against APP beta secretase 1 decreased amyloid plaque levels and neurodegeneration in APP transgenic mice [341]. Gene therapy has also been approved for SMA treatment as a one-time intravenous injection of onasemnogene abeparvovec, which introduces the SMN1 transgene into motor neurons to replace the non-functional SMN1 gene [342]. Moreover, the antisense oligonucleotide targeting the SMN2 gene nusinersen has also been approved. Both therapies have proved beneficial in the treatment of SMA [343,344]. A gene therapy approach has also been proposed in X-ALD using corrected hematopoietic stem cells CD34+, but this alternative treatment can only be applied to a small subset of patients with no severe symptoms [345]. 

## 6. Conclusions

In conclusion, oxygen presents a critical role in multiple metabolic and physiological processes in the human body, and its imbalance can lead to several pathological consequences, especially in the brain, which is highly sensitive to oxygen equilibrium. Dysfunctions caused by oxygen imbalance can result in hypoxia, hyperoxia, protein misfolding, mitochondria dysfunctions, alterations in heme metabolism and neuroinflammation, ultimately causing neurological alterations in both pediatric and adult life. These disorders share common pathways related to redox imbalance. With this review, we have highlighted the dysfunctions present in adult and pediatric neurodegenerative disorders, emphasizing their underlying redox dysfunction and summarizing the potential therapeutic strategies. Further research is still needed to explore these mechanisms and potentially identify therapeutic strategies effective in the treatment of these disorders.

## Figures and Tables

**Table 1:** Summarized pathological features related to oxidative stress in adult neurodegenerative disorders.

Pathological Feature	Alzheimer’sh Disease	Parkinson’sh Disease	Amyotrophich Lateral Sclerosis
Hypoxia	Aberrant amyloid β metabolism [97]	β-synuclein accumulationh [98]	/
Hyperoxia	Tau polymerizationh [99,100]	NADPH oxidase activation [101]	Increased markers for lipid and DNA oxidationh [102]
Heme proteinsh and iron	Decrease in hemoglobin transcription [50]	Altered iron metabolism, low SN hemoglobin [103]	/
Mithocondriah dysfunctions	Krebs cycle enzymes deficiencies [104]	Increased mitophagyh [105,106]	Impaired mitophagy, h altered mitochondria morphology [107,108]
Neuroinflammation	Microglia activationh [109]	Microglia activation, lymphocytes infiltrationh [110,111,112]	Inflammatory cytokine production [113]
Protein misfolding	Amyloid plaques formationh [114,115]	α-syn aggregationh [116]	Cytoplasmic inclusionsh [117]
